# Human cytomegalovirus infection-induced lymphocytosis diagnosed by metagenomic next-generation sequencing: a case report and literature review

**DOI:** 10.3389/fimmu.2025.1637085

**Published:** 2025-09-02

**Authors:** Shaofan Zhang, Li Sun, Liman Wang, Yuwei Liu, Gao Tan, Tao Liu, Wen Yang, Xiaoli Liu

**Affiliations:** Infection Department, Hunan Provincial Corps Hospital of Chinese People’s Armed Police Force, Changsha, China

**Keywords:** human cytomegalovirus, lymphocytosis, metagenomic next-generation sequencing, targeted next-generation sequencing, immunocompromised, case report

## Abstract

**Background:**

Human cytomegalovirus (HCMV) exhibits a high prevalence and is a major threat to immunocompromised individuals. Conventional diagnostic modalities are increasingly struggling to meet evolving clinical needs. Metagenomic next-generation sequencing (mNGS) represents a valuable tool for expeditious microbial identification in diagnostically complex cases.

**Case presentation:**

A 35-year-old man presented with fever, pharyngitis, fatigue, and marked lymphocytosis. No significant abnormalities were detected in imaging and routine tests, and conventional pathogen detection methods failed to identify any suspected pathogens. Targeted next-generation sequencing (tNGS) identified five pathogens: *Staphylococcus aureus*, *Streptococcus mitis* group, rhinovirus C, cytomegalovirus (CMV), and human herpesvirus-7. Clinical symptoms alleviated within 7 days of ganciclovir therapy initiation; however, lymphocytosis persisted. Subsequently, mNGS was performed, confirming HCMV infection and providing a definitive diagnosis. During follow-up, the patient’s symptoms had largely resolved.

**Conclusion:**

Symptomatic HCMV infections primarily affect immunocompromised individuals, while persistent lymphocytosis associated with HCMV is uncommon. This case highlights the diagnostic and therapeutic utility of mNGS in HCMV infection, especially when conventional diagnostic methods are limited, pathogen abundance is low, and the patient is immunocompromised.

## 1 Introduction

Human cytomegalovirus (HCMV), a beta-herpesvirus classified as human herpesvirus 5, has co-evolved with humans over millions of years. HCMV ranks among the most prevalent pathogens in clinical infectious diseases, and its large double-stranded linear DNA genome (230–240 kilobases) represents the most complex genetic architecture among human herpesviruses (1–3). It is highly contagious and widespread globally, with an estimated infection rate of approximately 70% worldwide (4). HCMV exhibits broad tissue tropism, with detectable virions in multiple biological matrices, including blood, saliva, urine, breast milk, and genital secretions. Its activity may persist for weeks or even years (5, 6). Most HCMV infections are asymptomatic, but a minority of cases present mild, self-limiting symptoms resembling mononucleosis-like conditions, including fever, fatigue, sore throat, muscle pain, joint pain, swollen lymph nodes, and enlarged liver or spleen (7). Standard diagnostic modalities encompass the following: 1) quantitative polymerase chain reaction (qPCR; HCMV DNA detection), 2) antigenemia assays, 3) immunohistochemical (IHC) analysis, 4) viral culture, 5) histopathological evaluation, and 6) nucleic acid hybridization (7, 8). Among these, qPCR (peripheral blood) has emerged as the diagnostic gold standard, offering superior sensitivity (98%) and specificity (95%) for active infection monitoring (7). However, qPCR detection of viral DNA requires careful interpretation, as positive results may represent latent viral reservoirs or residual genomic fragments from resolved infections. Transient viremic episodes (“blip phenomenon”) and inconsistent viral load thresholds (500–1,000 copies/mL) risk diagnostic misinterpretation (7). Additionally, interlaboratory variability in qPCR platforms (e.g., TaqMan vs. SYBR Green) and calibration standards (WHO vs. in-house) compromises result comparability (9).

Conventional diagnostic and therapeutic strategies increasingly struggle to address contemporary clinical demands. The emergence of novel technologies has triggered revolutionary transformations in clinical practice (10, 11). As a paradigm-shifting technology, metagenomic next-generation sequencing (mNGS) has revolutionized infectious disease diagnostics in recent years. mNGS enables direct analysis and characterization of microbial genetic material in clinical specimens via high-throughput sequencing. This culture-independent methodology provides unbiased pathogen detection without requiring prior microbial cultivation (12). The platform demonstrates concurrent detection capability for diverse pathogens (bacteria, viruses, fungi, and parasites) while maintaining diagnostic efficacy despite antibiotic pretreatment (13). Herein, we presented a rare case of HCMV infection-induced lymphocytosis where conventional diagnostics failed but mNGS achieved definitive identification.

## 2 Case presentation

A 35-year-old male patient was admitted to the Department of Infectious Diseases at our hospital at 8:55 AM on January 20, 2025. Eleven days prior to admission, the patient developed fatigue and pharyngitis, followed by recurrent fever after a 4-day interval. The patient had no significant medical history except for two coronavirus disease 2019 (COVID-19) infections in 2022 and 2023. Unremarkable epidemiological, personal, family, and allergy histories were noted. His 2024 annual physical examination revealed no special abnormalities. The initial symptoms of fatigue and pharyngitis emerged 11 days pre-admission without identifiable triggers, accompanied by absent fever or cough. He visited the outpatient department of our hospital, where a blood routine test revealed the following: white blood cells 10.96 × 10^9^/L (lymphocytes 3.97 × 10^9^/L, 36.2%; monocytes 1.66 × 10^9^/L, 15.1%; neutrophils 5.02 × 10^9^/L, 45.8%). A preliminary diagnosis of acute upper respiratory infection was made. Cefixime and Chinese patent medicines were administered with unsatisfactory therapeutic response. Seven days prior to admission, recurrent febrile episodes developed with a peak self-recorded temperature of 38.5°C. Apart from pharyngitis and fatigue, he reported no other symptoms. Self-administered oseltamivir and ibuprofen provided transient symptomatic relief. One day before admission, the patient revisited the outpatient department of our division with a body temperature of 38.1°C. Both influenza and COVID-19 antigen tests were negative following outpatient evaluation. Chest CT findings were unremarkable. C-reactive protein (CRP) levels were within the normal limits. Nevertheless, the blood routine test indicated the following: white blood cells 23.14 × 10^9^/L (lymphocytes 13.59 × 10^9^/L, 58.70%; monocytes 1.92 × 10^9^/L, 8.3%; neutrophils 7.29 × 10^9^/L, 31.50%). A comparison of the two routine blood tests showed lymphocyte absolute value escalation (3.97 → 13.59 × 10^9^/L), constituting the primary contributor to leukocytosis (10.96 → 23.14 × 10^9^/L). To determine the etiological factor precisely, the patient agreed to be hospitalized for further evaluation the next day.

On admission, the patient’s vital signs were as follows: body temperature 37.2°C, heart rate 106 beats/min, respiratory rate 18 breaths/min, and blood pressure 153/116 mmHg (1 mmHg = 0.133 kPa). Physical examination revealed no obvious superficial lymph node enlargement or abnormalities on cardiac and pulmonary auscultation. The main and persistent symptoms included fatigue, pharyngitis, and recurrent pyrexia. Given the marked leukocytosis and lymphocytosis, bone marrow aspiration and culture were performed on hospital day 2 to exclude hematological malignancies. Bone marrow analysis conducted at Changsha KingMed Center for Clinical Laboratory (KingMed Diagnostics, a College of American Pathologists (CAP)-accredited reference laboratory) demonstrated a hyperactive marrow pattern with a high proportion of lymphocytes (36% in bone marrow smear and 57% in blood smear). A comprehensive diagnostic workup was subsequently undertaken. Routine laboratory tests showed normal levels of procalcitonin (PCT), CRP, erythrocyte sedimentation rate (ESR), electrolytes, coagulation profile, and 12 tumor markers. However, hepatic function and myocardial enzyme levels were elevated: serum alanine aminotransferase (ALT) 276.5 U/L (ref 0–40 U/L), aspartate aminotransferase (AST) 121.3 U/L (ref 0–40 U/L), and lactate dehydrogenase (LDH) 492.8 U/L (ref 135–225 U/L). Cross-sectional imaging (head/chest/abdomen CT) was unremarkable, although cervical Doppler ultrasound detected bilateral subcentimeter lymph nodes (maximum 8 × 4 mm). Autoimmune serology (antinuclear antibody (ANA), antineutrophil cytoplasmic antibody (ANCA), rheumatoid factor (RF), etc.) and hematological markers (serum protein electrophoresis) were non-diagnostic. This excluded major rheumatologic (systemic lupus erythematosus, vasculitis, and rheumatoid arthritis) and hematological (multiple myeloma) disorders. Comprehensive microbiological workup included multiplex PCR panels, automated blood cultures, and serological assays for bacterial, fungal, parasitic, and viral pathogens. Except for Gram-positive bacteria detected in the bone marrow culture—suspected to be a contaminant—no pathogens were identified. Notably, two consecutive tests for Epstein–Barr virus (EBV) serology (VCA-IgM/IgG) and PCR remained negative.

Post-admission clinical manifestations included febrile episodes (peak 38.1°C) with associated pharyngitis, persistent fatigue, and mild anxiety disorder. The initial working diagnosis was fever of unknown origin (FUO), with viral etiology considered most likely. Accordingly, the initial antiviral regimen comprised ribavirin (500 mg IV BID) plus interferon α-1b (30 μg nebulized QD), supplemented with glutathione (1,200 mg IV QD) for hepatoprotection, and other supportive measures. Febrile episodes typically occurred in the afternoon and at night. Therapeutic intervention achieved temperature modulation (<38.0°C) within 72 hours. To guide further treatment, a nasopharyngeal swab was collected on January 24, 2025, and sent to Changsha KingMed Center for Clinical Laboratory for targeted next-generation sequencing (tNGS; a respiratory pathogen panel covering 225 pathogens). A positive result was obtained the following day: *Staphylococcus aureus*, *Streptococcus mitis* group, rhinovirus C, cytomegalovirus (CMV), and human herpesvirus-7. Based on the tNGS findings, ribavirin was replaced with ganciclovir (5 mg/kg every 12 h, IV infusion) on January 25, 2025, while other treatments remained unchanged. Ganciclovir therapy achieved afebrile status within 7 days, with progressive resolution of constitutional symptoms. Subsequently, all medications except ganciclovir were discontinued. A repeated blood routine test conducted on February 4, 2025, showed leukocytosis (24.71 × 10^9^/L) and lymphocytosis (17.55 × 10^9^/L). Although symptoms had resolved, leukocytosis and lymphocytosis persisted (exceeding admission values), with marked elevation of absolute lymphocyte count (13.59 → 17.55 × 10^9^/L).

To investigate persistent lymphocytosis, the patient, who was discharged on February 4, 2025, visited the Hematology and Infectious Diseases outpatient departments at Xiangya Hospital, Central South University, on February 6, 2025. A complete blood count (CBC) performed at Xiangya Hospital revealed leukocytosis (23.2 × 10⁹/L) with lymphocytosis (16.2 × 10⁹/L), consistent with our institutional findings. Peripheral blood smear analysis showed the following: neutrophils (28%), lymphocytes (60%), monocytes (6%), and atypical lymphocytes (6%). A comprehensive viral panel testing, including CMV PCR, returned negative results. Cervical color Doppler ultrasound identified bilateral hypoechoic nodules with regular morphology, well-defined borders, and heterogeneous echotexture. The largest nodules measured 26 × 10 mm (right, Level II) and 21 × 10 mm (left, Level II). Axillary regions contained similar nodules with maximal dimensions of 13 × 7 mm (right) and 14 × 6 mm (left). Nodules lacked internal vascularity. No pathologically enlarged lymph nodes were detected in the supraclavicular, subclavian, or inguinal regions. Lymphoma flow cytometry demonstrated the following: 1) T-cell predominance (59.4% of nucleated cells) and inverted CD4/CD8 ratio (0.19), and no obvious abnormalities were found in the other immunophenotypes; 2) NK cells comprised 5.1% and B cells 2.3% (predominantly mature polyclonal population); and 3) plasma cells constituted 0.1% of cells without clonal predominance. Based on the findings, the experts from the Hematology and Infectious Diseases departments of Xiangya Hospital concluded reactive lymphocytosis secondary to viral etiology, although the causative viral agent remained unidentified.

The patient was readmitted to our hospital on February 11, 2025, for diagnostic evaluation and management of persistent lymphocytosis. Guided by the Chinese expert consensus on mNGS in infectious diseases and through multi-disciplinary consultation, a peripheral blood sample was submitted to Changsha KingMed Center for Clinical Laboratory for mNGS on February 15, 2025. mNGS results returned the following day confirmed isolated cytomegalovirus infection. This definitive finding confirmed cytomegalovirus-associated lymphocytosis. Antiviral therapy with ganciclovir was maintained (5 mg/kg, q12h, initiated January 26), transitioning to daily maintenance dosing (5 mg·kg^−1^·day^−1^) after 3-week induction, with dose titration guided by clinical response and laboratory parameters. Serial CBC monitoring and lymph node surveillance were performed throughout hospitalization. Follow-up CBC on February 20 revealed WBC 12.09 × 10^9^/L (absolute lymphocytes 6.59 × 10^9^/L). These parameters demonstrated significant improvement from baseline, and the patient was discharged on February 21, 2025, with a stable clinical status.

**Figure 1:**
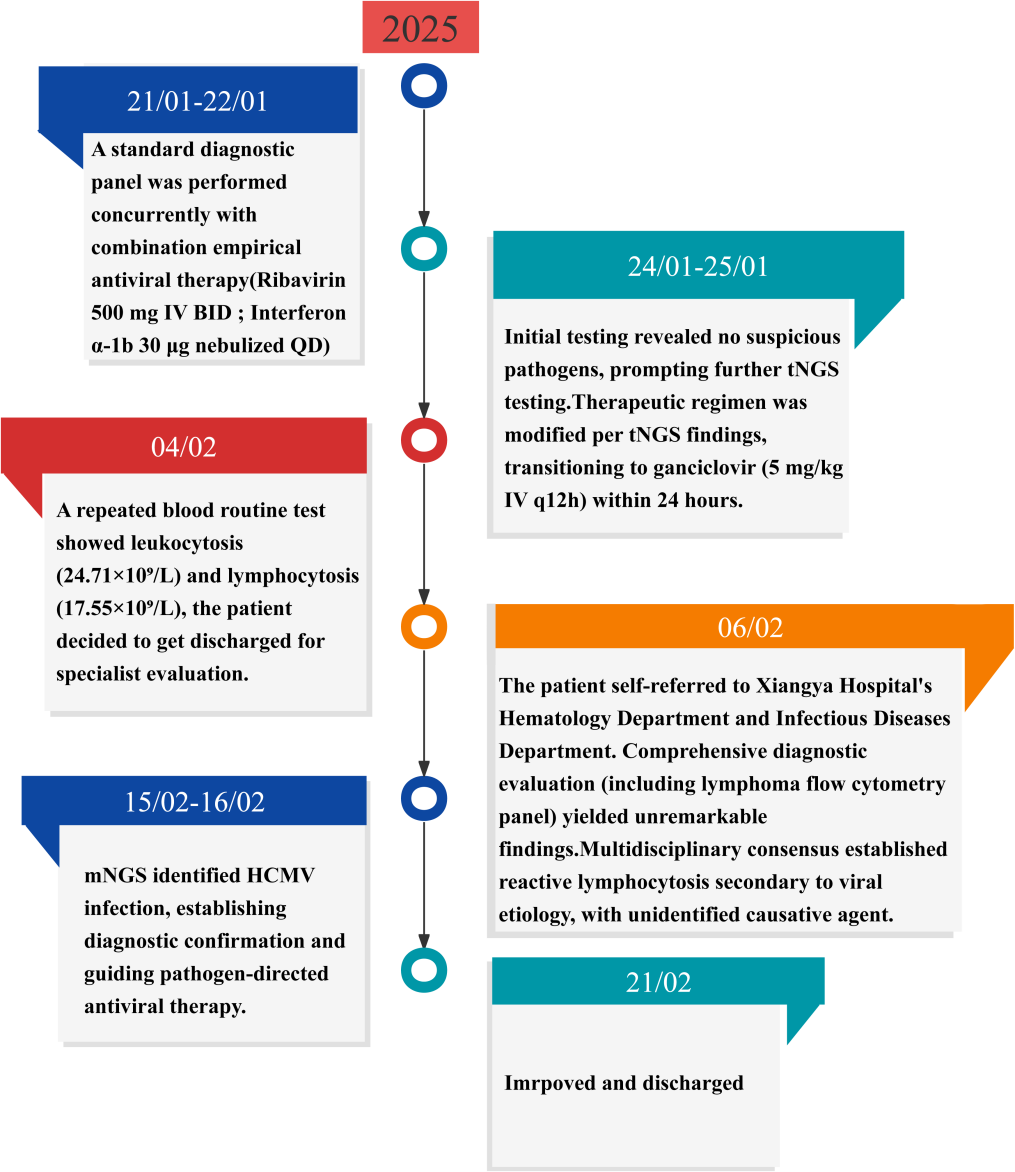
Timeline of disease progression and treatment. HCMV, human cytomegalovirus; mNGS, metagenomic next-generation sequencing; tNGS, targeted next-generation sequencing.

Following discharge, outpatient follow-up was continued. The patient discontinued antiviral therapy prematurely without medical guidance. At the penultimate follow-up (April 06, 2025), superficial lymphadenopathy resolved, liver function normalized, and the patient remained asymptomatic. However, peripheral blood abnormalities persisted (**Table 1**). The clinical constellation conclusively confirmed HCMV-associated infectious mononucleosis, supported by a characteristic hematological profile, virological confirmation via mNGS, and a therapeutic response pattern. mNGS results were instrumental in guiding therapeutic decisions and confirming the diagnosis. **Figure 1** graphically summarizes the diagnostic and therapeutic chronology, highlighting key decision points.

**Table 1:** CBC for the patient with HCMV.

Remarks	Dates	WBC (×10^9^/L) (ref 4–10)	NEU# (×10^9^/L) (ref 1.8–8.89)	Lym# (×10^9^/L) (ref 1.26–3.35)	Mon# (×10^9^/L) (ref 0.25–0.95)	NEU% (ref 41–74.3)	Lym% (ref 18.3–47.9)	Mon% (ref 4.2–15.2)
Pre-admission	01/11/25	10.96	5.02	3.97	1.66	45.80%	36.20%	15.10%
	01/19/25	23.14	7.29	13.59	1.92	31.50%	58.70%	8.30%
First admission	01/21/25	17.61	6.18	10.03	1.28	35.10%	56.90%	7.30%
	01/23/25	22.28	7.07	12.91	2.04	31.70%	57.90%	9.20%
	01/25/25	22.44	7.01	13.11	2.13	31.20%	58.40%	9.50%
Outpatient follow-up	02/04/25	24.71	5.59	17.55	1.38	22.60%	71.00%	5.60%
	02/06/25	23.2	5.4	16.2	1.3	23.20%	70.10%	5.50%
Second admission	02/12/25	15.25	5.58	8.82	0.64	36.60%	57.80%	4.20%
	02/18/25	14.5	5	8.6	0.7	34.20%	59.10%	5.00%
	02/20/25	12.09	4.64	6.59	0.59	38.30%	54.50%	4.90%
Outpatient follow-up	03/01/25	14.44	6.85	6.19	0.97	47.40%	42.90%	6.70%
	03/15/25	12.88	5.67	5.92	1.06	44.00%	46.00%	8.20%
	04/06/25	15.95	5.28	9.45	0.92	33.10%	59.20%	5.80%
	06/03/25	14.69	4.75	8.71	0.97	32.40%	59.30%	6.60%

WBC, white blood cell; NEU, neutrophils; Lym, lymphocyte; Mon, monocyte; HCMV, human cytomegalovirus; CBC, complete blood count.

## 3 Discussion

HCMV infections manifest in three forms: primary infection (no prior exposure), secondary infection (reactivation of latent virus), and reinfection with a different HCMV strain (14). The pathogenesis of HCMV is complex. Intricate interactions between HCMV and the host immune system lead to the development of immune evasion strategies that prevent recognition by immune cells (15–17). HCMV establishes lifelong latency in myeloid progenitor cells, with periodic reactivation occurring under immunosuppressive conditions (16, 17). Clinical manifestations of HCMV vary based on the host’s immune status and infection type. Congenital infection can cause severe neurological sequelae, whereas immunocompromised individuals may exhibit multi-organ involvement. In immunocompetent individuals, HCMV typically causes a mononucleosis-like syndrome (7).

**Figure 2:**
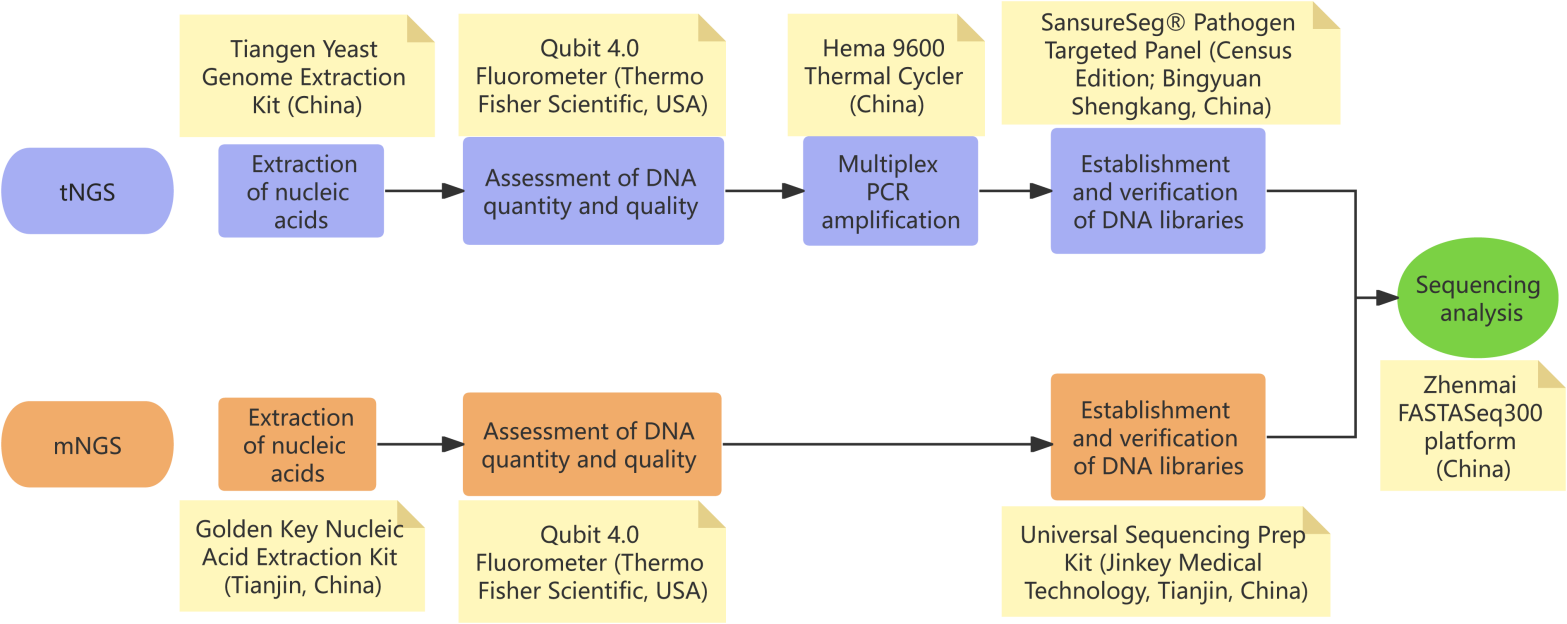
Technical roadmap of next-generation sequencing (NGS) workflows employed in our study. **(A)** Targeted NGS (tNGS): the validated limit of detection (LoD) is 100 copies/mL for the targeted microbial panel; organisms present at concentrations below this threshold may not be reliably detected. **(B)** Metagenomic NGS (mNGS): under the standardized condition of 1 × 10^5^ host cells/mL, the validated LoDs are 100 CFU/mL for bacteria, 500 CFU/mL for fungi, and 500 copies/mL for viruses; microbes present below these cut-offs may yield insufficient reads for confident pathogen calling.

In our case, the patient presented with mild non-specific symptoms, including fever, fatigue, pharyngitis, and peripheral lymphadenopathy. Differential diagnosis excluded hematological diseases, autoimmune conditions, and malignancies. Research shows that EBV constitutes the primary etiology of infectious mononucleosis, and HCMV serves as a less frequent causative agent, responsible for approximately 10% of cases (18). Therefore, lymphocytosis with atypical lymphocytes prompted the initial suspicion of EBV-associated infectious mononucleosis. However, repeated EBV serological and molecular assays returned negative results, as did HCMV testing performed externally. Definitive diagnosis of HCMV-induced infectious mononucleosis was achieved through the sequential application of tNGS and mNGS (**Figure 2** exhibits the NGS technical workflow implemented in this study). tNGS and mNGS differ in pathogen detection scope. mNGS, a non-targeted metagenomic sequencing technique, comprehensively identifies known pathogens, especially in patients with negative conventional tests but suspected infection (19, 20). tNGS provides targeted pathogen identification support. mNGS not only confirmed HCMV but also excluded co-infections, enabling definitive etiological determination.

In order to explore the connection between HCMV and lymphocytosis, a systematic literature search was conducted using PubMed and Web of Science (from 1966 to 2025). The search strategy included the following terms: (“Cytomegalovirus” OR “Salivary Gland Virus*” OR “Herpesvirus 5, Human” OR “HHV 5”) AND “Lymphocytosis”. The literature review (**Table 2**) suggested that HCMV infection-associated lymphocytosis is not rare (21–31). However, persistent lymphocytosis remains an uncommon clinical manifestation, documented in only two reported cases (23, 26) to date. The precise pathogenesis of this phenomenon has yet to be elucidated. In our case, the patient also exhibited persistent lymphocytosis (>4 months’ duration) with sustained elevation patterns. Lymphoma screening via flow cytometry at Xiangya Hospital revealed a CD4/CD8 ratio inversion (0.6), indicating that the patient had partial immunosuppression and impaired pathogen clearance capacity. Symptomatic HCMV infection typically results from the reactivation of latent virus, often as an opportunistic infection in immunocompromised individuals (17). Therefore, the observed clinical course above suggests recurrent HCMV reactivation following immunosuppression, potentially indicating chronic viral persistence. Despite the established sensitivity of qPCR for HCMV detection (7), serial testing yielded negative results in our case. Only through NGS could its presence be detected. Thus, all these findings suggest that there may be a chronic low-grade HCMV infection with subthreshold viral loads in this patient. A previous study has shown that chronic HCMV infection may cause persistent lymphocytosis—particularly CD8+ T-cell expansion—that can last from months to years (32). The precise mechanisms remain elusive, but we think that this chronic viral persistence may induce immune remodeling that sustains lymphoproliferation. Notably, this immunocompetent young man lacked traditional risk factors for chronic viral persistence. However, the cause of his apparent immune dysfunction remained unclear. His medical history revealed two COVID-19 infections (2022 and 2023) despite previous good health. The post-infection sequelae included new-onset fatigue susceptibility and recurrent upper respiratory infections. Therefore, it is plausible that his immune dysfunction was related to immune dysregulation or aberrant immune responses induced by prior COVID-19 infection (33, 34). Of course, factors like chronic fatigue (35), circadian rhythm disruption (36), and physical inactivity (37) may also have contributed.

**Table 2:** Clinical characteristics of 10 patients with HCMV-associated lymphocytosis.

References		Age	Sex	Underlying disease	Symptom	WBC#	Lym# or Lym%	Other pathogens	Infection therapy	Duration of lymphocytosis	Outcome
Patient 1	Matias-Lopes et al. (28)	62	Female	NA	Fatigue, generalized inflammatory arthralgias, hypogastric discomfort, and daily persistent fever (maximum 39.9°C)	17.6 × 10^9^/L	10,000/μL	Negative	Valganciclovir, corticosteroid	NA	Cured
Patient 2	Eirís et al. (26)	27	Male	Hepatosplenic T-cell lymphoma	Fever, hyperhidrosis	NA	15,550/μL	Negative	NA	>6 months	NA
Patient 3	Gonçalves et al. (27)	29	Male	Normal	Fever, headache, malaise, dry non-productive cough, and thoracic pleuritic pain	6,620/μL	48%	Negative	Ganciclovir	NA	Cured
Patient 4	Thamcharoen et al. (30)	75	Female	Normal	Altered mental status, septic picture, and influenza-like symptoms	13,200/μL	33%	Negative	NA	NA	NA
Patient 5	Crespo et al. (25)	31	Male	Lymphocytic meningitis, post-LP syndrome	Severe occipital headache, fever (39°C), dysesthesia of the scalp, and odynophagia	NA	55%	*Rickettsia conorii*, *HSV-1*	Ganciclovir	NA	Cured
Patient 6	Torres et al. (31)	23	Male	Splenectomized and polytransfused for a severe blunt trauma 10 months ago	Systemic inflammatory response syndrome	44,300/μL	NA	Negative	Ganciclovir	NA	Cured
Patient 7	Shimojima et al. (29)	43	Female	Rheumatoid arthritis	Intermittent high fever, general fatigue, and appetite loss	14,320/μL	46%	Negative	No specific treatment	NA	Cured
Patient 8	Sankar et al. (23)	15	Male	Systemic lupus erythematosus	Recurrent fever, easy fatigability, loss of appetite, failure to thrive, recurrent oral ulcers, and photosensitive rash	50,900/μL	95%	Negative	Ganciclovir	>9 months	Cured
Patient 9	Assy et al. (24)	36	Male	Splenectomy for blunt trauma	Fever	35,000/μL	70%	Negative	Ganciclovir	NA	Cured
Patient 10	Our case	35	Male	Normal	Fatigue, pharyngitis, and recurrent pyrexia	23.14 × 10^9^/L	13.59 × 10^9^/L	*Staphylococcus aureus*, *Streptococcus mitis* group, rhinovirus C, and human herpesvirus-7	Ganciclovir	>4 months	Cured

HCMV, human cytomegalovirus; NA, not available.

Currently, the clinical integration of mNGS for diagnosing and managing HCMV infections has not yet been established as a standard recommendation in major domestic and international guidelines. However, ongoing technological advancements and accumulated clinical experience have highlighted the diagnostic utility of mNGS in specific clinical scenarios, particularly for cases where conventional methods are limited, infections are polymicrobial, or patients are immunocompromised. Artificial intelligence (AI) has emerged as a prominent global research focus, with significant advancements in medical applications being particularly notable in recent years (38–40). Recent studies have demonstrated that the integration of next-generation sequencing with machine learning algorithms represents a paradigm-shifting approach for enhancing diagnostic accuracy and therapeutic decision-making (41). Hence, the synergistic development of mNGS and AI technologies holds substantial promise for overcoming current methodological constraints, particularly through enhanced data interpretation and analytical precision.

This research possesses certain limitations. First, the small sample size inherent to this single-case report design limits the generalizability of the findings. Therefore, cautious interpretation of these observations is warranted. Second, while flow cytometric analysis for lymphoma was performed at Xiangya Hospital, institutional technical constraints precluded definitive diagnostic procedures, including bone marrow biopsy and lymph node aspiration. Consequently, comprehensive exclusion of hematological malignancies remained diagnostically unconfirmed. Third, due to the rarity of the condition, our treatment approach did not yield a curative outcome and should be considered for reference only. This experience highlights the necessity for evidence-based guidelines to optimize therapeutic approaches for complex HCMV infections.

## 4 Conclusion

We present a rare case of persistent lymphocytosis induced by HCMV infection confirmed via mNGS. HCMV demonstrates ubiquitous seroprevalence in global populations, with the majority of infections remaining clinically silent. While symptomatic presentations predominantly affect immunocompromised hosts, sustained lymphocytosis represents an uncommon clinical sequela of particular diagnostic significance. This case underscores mNGS’s diagnostic superiority in three clinical contexts: 1) limitations of conventional assays, 2) low-abundance pathogen environments, and 3) immunocompromised patient populations. Technical refinements coupled with cost-optimized workflows position mNGS as an emerging cornerstone technology for pathogen detection and precision antimicrobial stewardship.

## Data Availability

The datasets presented in this study can be found in online repositories. The names of the repository/repositories and accession number(s) can be found in the article/supplementary material.
